# ON-1 and BA-IX Are the Dominant Sub-Genotypes of Human Orthopneumovirus A&B in Riyadh, Saudi Arabia

**DOI:** 10.3390/genes13122288

**Published:** 2022-12-05

**Authors:** Rasha M. Alzayed, Ibrahim M. Aziz, Asma N. Alsaleh, Gani Asa Dudin, Anwar A. Ahmed, Tajamul Hussain, Abdullah K. Alshememry, Ali M. Somily, Muslim M. Alsaadi, Fahad N. Almajhdi

**Affiliations:** 1 Department of Botany and Microbiology, College of Science, King Saud University, Riyadh 11451, Saudi Arabia; 2 Biology Department, College of Science, Jouf University, Sakaka 41412, Saudi Arabia; 3 Center of Excellence in Biotechnology Research, College of Science, King Saud University, Riyadh 11451, Saudi Arabia; 4 Department of Pharmaceutics, College of Pharmacy, King Saud University, Riyadh 11451, Saudi Arabia; 5 Department of Pathology, College of Medicine, King Saud University, Riyadh 11451, Saudi Arabia; 6 Department of Pediatrics, College of Medicine, King Saud University, Riyadh 11451, Saudi Arabia

**Keywords:** human *orthopneumovirus*, ON-1, BA-IX, 2nd HVR, HRSV, Riyadh, Saudi Arabia

## Abstract

*Human orthopneumovirus* (HOPV) is the major viral pathogen responsible for lower respiratory tract infections (LRTIs) in infants and young children in Riyadh, Saudi Arabia. Yet, predominant HOPV subtypes circulating in this region and their molecular and epidemiological characteristics are not fully ascertained. A total of 300 clinical samples involving nasopharyngeal aspirates (NPAs), throat swabs, and sputum were collected during winter seasons of 2019/2020 and 2021/2022 for HOPV subtyping and genotyping. Of the 300 samples, HOPV was identified in 55 samples (18.3%) with a distinct predominance of type A viruses (81.8%) compared to type B viruses (18.2%). Importantly, the ON1 strain of HOPV-A and BA-IX strain of HOPV-B groups were found to be responsible for all the infections. Sequence analysis revealed a duplication region within 2nd HVR of G protein gene of ON1 and BA-IX strains. This nucleotide duplication exerted a profound effect on protein length and affinity towards cell receptors. Further, these modifications may aid the HOPV in immune evasion and recurrent infections. Data from this study showed that ON-1 genotype of HOPV-A and BA-IX genotype of HOPV-B were dominant in Riyadh, Saudi Arabia. Further, a duplication of sequence within 2nd HVR of G protein gene was found.

## 1. Introduction

*Human orthopneumovirus* (HOPV), previously known as human respiratory syncytial virus (HRSV), belongs to the genus *Orthopneumovirus* within the family *Pneumoviridae* and order *Mononegavirales* [1]. It is a major viral pathogen causing LRTIs in infants and young children [2,3]. Globally, HOPV is responsible for over 30 million LRTIs per year and approximately 3.4 million hospitalizations with about 60% of episodes occurring in children below 5 years old [4,5].

HOPV is an enveloped virus with a 15.2 kb negative-sense single-stranded RNA genome containing 10 genes and encodes 11 proteins [6]. The G protein helps in virus attachment and entry into the host cell [7]. By virtue of antigenic variations found in the G glycoprotein and interactions with monoclonal antibodies (mAbs), HOPV strains are classified into two main antigenic subgroups, A and B [8,9]. Although HOPV-A and B co-circulate during the epidemic type A is slightly more prevalent than B [10]. We have previously shown that in Saudi Arabia, HOPV-A and B are major circulating subtypes with a slight predominance of HOPV-A responsible for lower respiratory tract infection in hospitalized children (19.3–45.4%) [11,12,13].

Based on the gene sequence variability, antigenic groups can be further classified into C-terminal region of 2nd HVR of G protein gene [14]. A number of genotypes representing HOPV have been documented including SAA1, CB-A, ON1, GA1 to GA7, and NA1 to NA4, genotypes of HOPV-A and URU1 and URU2, BA1 to BA10, BA-C, THB, GB1 to GB4, and SAB1 to SAB4 of HOPV-B [6,15]. Of importance is the BA genotype of HOPV-B, which was first detected in Buenos Aires (BA), Argentina, in 1999, and is characterized by a 60-nucleotide duplication in the 2nd HVR of G gene [16]. A similar duplication of 72-nucleotides in the 2nd HVR of the subtype ON1 of HOPV-A was detected in Canada [17]. In countries such as Malaysia, India, South Korea, Germany, Italy, South Africa, Japan, China, Kenya, Iran, and Saudi Arabia, the ON1 genotype appears to be gaining predominance over the previously predominant NA1 genotype [18,19,20,21,22,23,24,25].

The molecular epidemiology and circulation pattern of HOPV in Saudi population is incompletely understood. We have previously reported that all of the Saudi HOPV-B strains clustered in the BA-IX genotype [12]. We also reported a genotype shift from NA1 to ON-1 in HOPV-A, which was prevalent during the winter seasons of 2007/2008 and 2008/2009 [12,13]. Here, we examined predominant HOPV subtypes and their genotype variation in the clinical samples collected during two winter seasons of 2019–2020 and 2021–2022 in Riyadh.

## 2. Materials and Methods

### 2.1. Sample Collection

A total of 300 clinical samples involving nasopharyngeal aspirates (NPAs), throat swabs, and sputum were collected from all the children including infants and young children under 5 years old of both sexes admitted to King Khalid University Hospital (KKUH), Riyadh, Saudi Arabia with symptoms including cough, rhinorrhea, dyspnea, and fever. The assessment of severity was based on the number of days the hospitalized patient was on supplemental oxygen. Patients with ≥2 days on oxygen were classified as having severe illness, while those on oxygen for a single day or without shortness of breath were classified as having a mild infection. Clinical samples during the winter season (October to January) of 2019/2020 (*n* = 250) and 2021/2022 (*n* = 50) were obtained following the protocols approved by the Ethical Committee of King Saud University (Ethics Reference No. E-141326/IRB) and after obtaining written informed consent from the parents/guardians of the patients. Due to COVID-19 pandemic-related lockdown, we could not collect samples during the 2020–21 winter season. Immediately after collections, samples were mixed with 2 mL of the virus transport medium (PBS) and transported on ice and stored at −80 °C until analyzed.

### 2.2. Detection, Typing, and Sequencing of HOPV

Viral RNA from clinical samples was extracted using QIAamp Viral RNA Extraction Kit (Qiagen, Hilden, Germany). Presence of HOPV RNA in the extracted RNA was determined using OneStep RT-PCR Kit (Qiagen, Hilden, Germany). Using the same kit, HOPV-A and B typing was performed by amplifying the G gene. The reaction was conducted in a GeneAmp 9700 thermal cycler. Reverse transcription was performed at 50 °C for 30 min followed by initial denaturation at 95 °C for 15 min and 35 cycles of amplification each consisting of denaturation at 94 °C for 30 s, annealing at 52 °C for 30 s, and extension at 72 °C for 2 min. Final extension was performed at 72 °C for 10 min. The sizes of amplified products were determined by agarose gel electrophoresis along with appropriate molecular weight marker (Qiagen, Hilden, Germany). Primer sequences used for the detection and typing of the HOPV are presented in Table 1.

Amplification of 2nd HVR of the G protein was performed in a GeneAmp 9700 thermal cycler using OneStep Ahead RT-PCR Kit with Taq High Fidelity DNA Polymerase (Qiagen, Hilden, Germany). Primer sequences used in PCR are presented in Table 1. From the pool of positive samples, 9 samples from group A and 5 from group B were sequenced to represent the entire positive sample. The amplified fragments of the 2nd HVR of the G protein gene were sequenced on both strands at Macrogen Inc (Seoul, South Korea). DNA sequence editing was performed using Bioedit program, version 7.0 (Ibis Biosciences, Carlsbad, CA, USA). The final sequences were deposited in GenBank with accession numbers OP554434–OP554447.

### 2.3. Sequence Data and Phylogenetic Analysis

To trace the evolution of Saudi HOPV-A and B in Riyadh at multiple sequences, both nucleotides and deduced amino acid sequences were aligned using Clustal W, (of MegAlign program of Lasergene software, version 3.18 (DNAStar, Madison, WI, USA). Identification of mutation loci, divergence analysis, and prediction of AA changes were performed using EditSeq and MegAlign programs, and Lasergene software, version 3.18 (DNAStar Inc., Madison, WI, USA). Sequence variants were identified by comparing with GenBank sequences of strains of different HOPV-A genotypes (Tables S1 and S2: List of HOPV-A and HOPV-B strains included in sequence and phylogenetic analysis). Phylogenetic analysis was performed using MEGA 7 (v.11, Pennsylvania State University, University Park, PA, USA). The accuracy of the tree topology was evaluated by bootstrapping 1000 replicates.

The prediction of potential N-and O-linked glycosylation sites in G-protein of HOPV-A and B strains were assessed using Net-N-glyc 1.0 (http://www.cbs.dtu.dk/services/NetNGlyc) accessed on 29 September 2022 and Net-O-glyc 3.1 (http://www.cbs.dtu.dk/services/NetOGlyc), accessed on 29 September 2022 respectively.

## 3. Results

### 3.1. Prevalence of HOPV

HOPV was identified in 55 samples (18.3%) of the 300 samples with a distinct predominance of type A virus (45;81.8%) compared to type B virus (10;18.2%). The demographic and clinical information of the patients and distribution of positive results in two epidemic seasons is shown in (Table 2). Infants <1 year of age were the most affected age group, comprising (58.2%) of the total number of cases. HOPV was predominant in males (63.6%) compared to females (36.4%) from the total number of cases.

### 3.2. Genetic Analysis of HOPV Strains

**Figure 1:**
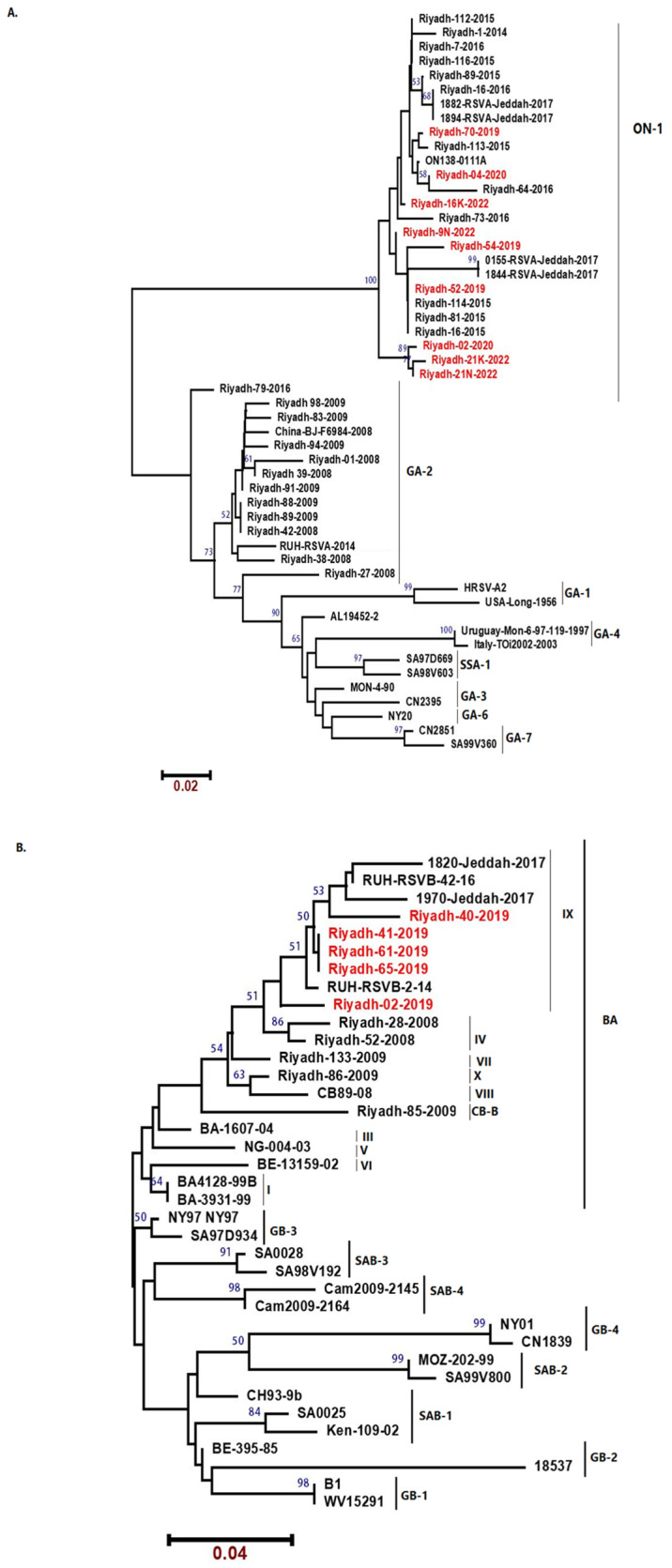
Phylogenetic trees for HOPV group A (**A**) and group B (**B**). The 270 base nucleotide sequences of the C-terminal fragment of the 2nd HVR of the G protein gene were used for alignment. The phylogeny tree was created using the neighbor-joining method using MEGA11 program. The numbers at the internal nodes of the tree represent the bootstrap values of 1000 replicates. Only values exceeding 60% are shown. Strains of seasons 2019/2020 and 2021/2022 are denoted by red.

Phylogenetic analysis constructed by the neighbor-joining method for the 9 HOPV-A positive samples with other reference sequences from GenBank showed that all 9 studied HOPV-A strains belonged to the ON1 genotype (Figure 1A). In addition, phylogenetic analysis of study HOPV-B strains sequences showed that all five studied HOPV-B strains belonged to the BA-IX genotype (Figure 1B).

### 3.3. Amino Acid Sequence Analysis of the G Gene

**Figure 2:**
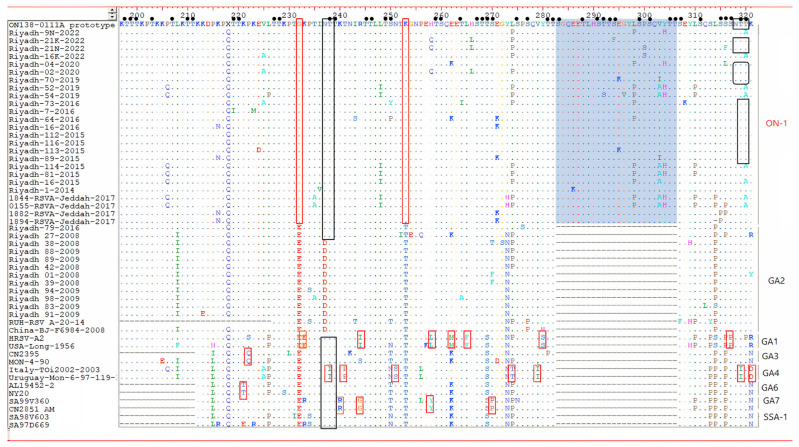
Alignment of deduced AA sequences of the 2nd HVR of the G genes of HOPV group A. The AA positions corresponded to AA positions of the relative to the sequences of prototype ON1 strain. Different AAs are marked in different colors, identical residues are presented by dots. The original and duplicated amino-acid region are shown in the green rectangles. Red rectangles color = the AA residues specific to genotype. Predicted N-glycosylation sites are enclosed in black rectangles. Small filled circles correspond to predicted O-glycosylation sites.

**Figure 3:**
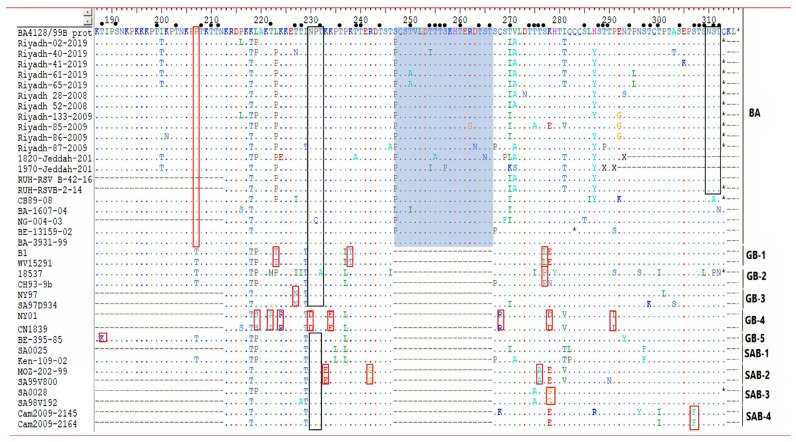
Deduced AA sequence alignment of 2nd HVR of G protein gene of the HOPV-B. The alignment was performed by the Clustal W method running within the MegAlign program (DNAstar). Alignment is shown relative to the BA prototype strain from Argentina. Dots are indicated for identical AA residues, while sequence variation is identified in single-letter code. The duplicated regions are shown in the green rectangles. Red rectangles color = the AA residues specific to HOPV-genotype. Predicted N-glycosylation sites are enclosed in black rectangles. Small filled circles = predicted O-glycosylation sites.

Deduced AA sequences of the 2nd HVR of the G genes of HOPV group A and B are presented in Figure 2 and Figure 3. Analysis of the last C-terminal AA residues of the G protein, 110 AA for ON-1 with a TGA as a stop codon similar to the prototype ON1 strain (ON138-0111A) from Canada and 86 AA for the other genotypes, has revealed 21 conserved residues. The studies on ON-1 strains have similar AA that were assigned to individual genotypes 232 G and 253 K.

### 3.4. N- and O-Glycosylation Site Analysis in Amino Acid Sequence

The N- and O-glycosylation site analysis in amino acid sequence at the C-terminal region of the G protein of Saudi strains showed three to seven sites in the investigated HOPV-A sequences. The N-glycosylation sites of the ON-1 subgenotype, including the HOPV-A strains identified in this study, presented only 3 conserved N-glycosylation sites at residues at aa 103 (NLS), 135 (NTT), and 237 (NTT). The first two sites are conserved in all HOPV-A strains included in this study. Another site was identified in the majority of Saudi strains, including residues 318 (NTT), found in the majority of HOPV-A genotypes. Whereas, for the investigated HOPV-B sequences, two to four N-glycosylation were observed. Glycosylation at residues 296 and 310 (NST) were found in the majority of HOPV-B genotypes sites as predicted in the sequences of the samples. The first N-glycosylation located at residue 230 (NPT) was conserved among all HOPV-B genotypes except GB4.

The number of O-glycosylation sites at serine and threonine residues in the C-terminal region of the G protein of the investigated HOPV-A sequences varies from 62 to 87 as determined by Net-Oglyc 3.1, whereas for HOPV-B, the number of O-glycosylation sites varies from 37 to 44.

## 4. Discussion

In the present study, HOPV was identified in 55 (18.3%) of the 300 samples with a distinct predominance of type A viruses (45;81.8%) compared to type B viruses (10;18.2%) among hospitalized children aged <5 years old during 2019/2020 and 2021/2022 in Riyadh. These observations are consistent with our previous study where out of 175 samples analyzed, 39 (22.3%) were positive for HOPV (59%) type A and (41%) type B in Riyadh during the winter-spring seasons of 2007/2008 and 2008/2009 [12]. Furthermore, we also reported that HOPV was found in 89 of 205 (43.4%) samples of which 56 (27.3%) were type A and 33 (16.1%) were type B viruses during the winter seasons of 2014/2015 and 2015/2016 [13]. In agreement with these findings, the prevalence of HOPV was between 0.2–54% in the children during 1991–2015 in Saudi Arabia [29]. A previous study also reported that HOPV was identified in the 26% of the hospitalized children in Riyadh between October and December, 2014. HOPV-A predominated (77%) during the study as compared to HOPV-B (23%) [30]. Furthermore, 50% of the NPAs were positive for HOPV with predominance of HOPV-A (72%) as compared to HOPV-B (24%) during the study in Riyadh during 2008–2016 [31].

In Jeddah, Saudi Arabia, HOPV represented 84/834 (13.4%) in the recruited cases with 64% of them belonging to group A and 36% to group B during January to December, 2017 [25]. Similarly, HOPV has reported in many countries; in Germany, HOPV was found to be 134/240 (55.4%) with a predominance of type A viruses 110 (82.1%) than type B viruses 24 (17.9%) [32]. In Madrid, Spain, out of 3011 respiratory samples, HOPV was detected in 640 (21.3%) and 405 were HOPV-A (63.2%) [33]. In Kuwait, HOPV was detected in 77 (25.2%) out of 305 samples, HOPV-A was predominant in 52 (67.5%) of the positive samples over group B viruses 25 (32.5%) of the positive samples [34]. In Lebanon, HOPV was detected in 16% (83/519) of the NPAs collected during the 2016/2017 season tested positive for HOPV; 50% (27/54) were HOPV-A and 50% (27/54) were HOPV-B [35].

In the present study, genotype ON1 strains remained the dominant HOPV-A genotype in two epidemic seasons (2019/2020 and 2021/2022). These observations, are in parallel with our previous study where majority of HOPV-A strains were found to be that of ON-1 sub-genotype in Riyadh during winter seasons 2014/2015 and 2015/2016 [13]. An earlier study reported that 26% of the positive samples clustered in ON-1 sub-genotype in Riyadh between October and December, 2014 [30]. A recent study reported that 97% of HOPV-A sequences (*n* = 28) clustered into ON1 genotype in Riyadh, during 2016 [31]. In a similar fashion, a recent study reported that all HOPV-A strains belonged to the ON-1 sub-genotype in Jeddah, Saudi Arabia [25]. The ON-1 was originally identified in 2010 in Ontario, Canada. ON1 is characterized by deletion of three consecutive nucleotides (852–854) and 72-nucleotides duplication at 2nd HVR of G protein gene. Such an insertion resulted 72-nt insertion in G, resulting in 24 extra Aas, of which 23 are duplications of 261 to 283 [17]. ON-1 has replaced the previously predominant NA1 genotype and is reported to be rapidly spreading in many countries (e.g., Germany, Italy, and South Korea Malaysia, India, South Africa, Japan, China, and Kenya) [18,19,20,21,22].

The rapid swap to the ON-1 strain may be explained by the presence of a 23 AA duplicated region in ON-1 strains resulting from novel antigenic features in the duplication segment that may be associated with higher rates of hospitalization and infection [23]. However, the correlation between the genotype and the severity of the disease is still unclear [10,36,37].

This study also revealed that circulating HOPV-B strains belonged to the BA-IX sub-genotype. Similarly, we reported that all HOPV-B strains belonged to the BA genotype in Riyadh during the winters of 2008 and 2009 [38]. An earlier study reported that 26% of the positive samples clustered in BA genotypes in Riyadh during between October and December, 2014 [30]. A previous study reported that all the HOPV-B sequences (*n* = 5) were in BA genotype in Riyadh, during 2016 [31]. The BA genotype was also reported from Jeddah, Saudi Arabia in a recent study [25]. The BA genotype is prevalent worldwide and has been reported in many countries including Kenya [39], Japan [40], Belgium [41], China [42], Brazil [43], Iran [44], Korea [45], and India [46].

The BA genotype is characterized by a 60-nucleotide duplication (generating a 20-aAs insertion in the C-terminal region) starting after residue 791 at the C-terminal in the 2nd HVR in the G gene [16]. The BA genotype has been further subdivided into subgroups (BA-I to BA-X) [47,48] and CB-B [45]. Subgroup BA-I, and II were predominant during the years 2002, and 2005, respectively. Whereas, in the years 2004–2008 BA-IV was more prevalent but was replaced, to a significant extent, by subgroups VII-X [47]. In this study, the phylogenetic analysis demonstrated that clustered HOPV-B strains in BA-IX genotypes. Sequence and phylogenetic analyses have revealed a sub-genotype shift from BA-IV, BA-CB-B, and BA-X to BA-IX, which was prevalent during the winter seasons of 2007/2008 and 2008/2009 [38]. The shift to the BA strain may be explained by the presence of 20- AA duplication region in BA strains, which may be associated with improving virus survival in nature [49].

In the current study, a significant homogeneity in the last C-terminal AA residues of the G protein, 110 AA for ON-1, and the usage of stop codon (TGA) was demonstrated. It showed similarity to prototype ON1 strain (ON138-0111A) from Canada. The AA analysis for HOPV-A in the 24 AA duplicated region revealed that most Saudi strains (contained two AA substitutions (L298P and Y304H) that were described earlier in Saudi strains (Riyadh-16-2015, Riyadh-73-2015, Riyadh-114-2015, Riyadh-64-2016, Riyadh-81-2015, 1844-HOPV -Jeddah-2017, and 0155-RSVA-Jeddah-2017). In addition, analysis of the deduced AA sequence of HOPV-B, all five study Saudi strains belonged to the BA subgroup that is characterized by 20- AA duplication. All Saudi trains identified in 2019/2020 had a TAA as a stop codon with a length of 312 AA. It is four AA shorter than the prototype BA strain from Argentina (316 AA). The AA analysis for HOPV-B in the 20 AA duplicated region revealed that all Saudi strains had AA substitutions (S247P) that were described earlier also in Saudi strains (Riyadh-28-2008, Riyadh-52-2008, Riyadh-85-2009, Riyadh-86-2009, Riyadh-133-2009, 1820-RSVA-Jeddah-2017 and 1970-RSVA-Jeddah-2017, RUH-RSV_B-42-16, and RUH-RSVB-2-14).

The insertion of AA in 2nd HVR of the G protein of HOPV resulted in a robust effect on the G protein in terms of functionality, glycosylation, and length. Analyzing the O-glycosylation sites at the C-terminal region of 2nd HVR of the G protein of Saudi strains showed (3–7) sites in the investigated HOPV-A sequences. The insertion of 24 AA in ON-1 sub-genotype led to addition of 11 new O-glycosylation sites (283S, 288T, 291S, 292T, 293T, 294S, 299S, 301S, 305T, and 306T) in the duplicated region, and 9 new O-glycosylation sites in BA sub-genotype (249S, 250T, 254T, 255T, 256T, 257S, 260T, 264T, 265S, and 266T). In contrast, the N-glycosylation sites of the ON-1 subgenotype or BA sub-genotype are limited, and duplication has no effect on the number of N-glycosylation sites. Variations in the N- and O-linked glycosylation of the G protein are an important landmark of antigenicity of the HOPV strains that may affect the expression of epitopes and contribute to immune evasion by either masking, facilitating, or influencing the antibody recognition [50].

This study was limited by the small sample size and its cross-sectional nature. Further study cannot suggest any cause for recurrent infections. Additional comprehensive investigations with a larger sample size covering different regions of Saudi Arabia in consecutive epidemic seasons are warranted for a more comprehensive understanding of HOPV circulation.

In conclusion, we presented here the genetic diversity of both HOPV-A and B in the G protein gene in Riyadh during the winter seasons of 2019/20 and 2021/22. This study showed that ON-1 genotype of HOPV-A and BA-IX genotype of HOPV-B with duplication in G protein gene were predominant in Riyadh during 2019–2022. The nucleotide duplicated region in 2nd HVR of the G gene of HOPV led to an immense effect on the G protein in terms of length, glycosylation, and functionality. The heterogeneity in the glycosylation in the N- and O-linked glycosylation profile of the G protein may contribute to immune evasion and cause recurrent infections. Our results also showed the predominance of the ON-1 genotype of HOPV-A and BA-IX genotype of HOPV-B within Riyadh. Further, comprehensive investigation involving samples from different regions of Saudi Arabia in consecutive epidemic seasons are required for understanding the evolutionary pattern of these emerging HOPV.

## Figures and Tables

**Table 1:** List of HOPV primers used in this study.

	Virus	Primer Name	Sequence	Amplicon Size (bp)	Ref.
For HOPV detection	HOPV	HOPV-U-F	5′-GGAACAAGTTGTTGAGGTTTATGAATATGC-3′	278	[26]
		HOPV-U-R	5′-CTTCTGCTGTCAAGTCTAGTACACTGTAGT-3′		
For HOPV typing	HOPV-A	HOPV A-F	5′-GATGTTACGGTGGGGAGTCT-3′	334	[27]
		HOPV A-R	5′-GTACACTGTAGTTAATCACA-3′		
	HOPV-B	HOPV B-F	5′-AATGCTAAGATGGGGAGTT-3′	183	
		HOPV B-R	5′-GAAATTG AGTTAATGACAG-3′		
For sequencing	HOPV-A	HOPVA-G-F2	5′-CAAGATGCAACAAGCCAGATC-3′	863	[12]
		HOPV-G-R2	5′-ACTGCACTGCATGTTGATTG-3′		
	HOPV-B	HOPVB-G-F2	5′-CCTTACTCAAGTCTCACCAGAAAG-3′	704	[28]
		HOPV-G-R2	5′-CTGTGGATCAGCAACTCCATG-3′		

**Table 2:** The demographic and clinical information and the prevalence of HOPV.

Seasonal and Demographic Details		Number of Samples	Number of Positive Samples (%)	Seasonal HOPV	
				HOPV-A (%)	HOPV-B (%)
Winter season	2019–2020	250	40 (16)	30 (75)	10(25)
	2021–2022	50	15 (30)	15 (100)	0
	Total	300	55 (18.3)	45 (81.8)	10(18.2)
Age in years	<1	115	32 (27.8)	18 (56.2)	7(70)
	1–2	78	12 (15.4)	13 (28.8)	2(20)
	3–4	54	9 (16.6)	7 (15.6)	1(10)
	≥5	53	2 (3.8)	7 (15.6)	-
Gender	Male	166	35 (63.6)	28 (62.2)	6(60)
	Female	134	20 (36.4)	17 (37.8)	4(40)
Severity	Mild	43	43 (78.2)	33 (73.3)	10(100)
	Severe	12	12 (21.8)	12 (26.7)	0

## Data Availability

All the data are provided.

## Supplementary Materials

The following supporting information can be downloaded at: https://www.mdpi.com/article/10.3390/genes13122288/s1, Table S1: List of HOPV-A strains included in sequence and phylogenetic analysis; Table S2: List of HOPV-B strains included in sequence and phylogenetic analysis.
