# Supplementary material for: ON-1 and BA-IX Are the Dominant Sub-Genotypes of Human Orthopneumovirus A&B in Riyadh, Saudi Arabia
Source: Genes (Basel). 2022 Dec 5;13(12):2288. doi: 10.3390/genes13122288 (PMC9778264; doi:10.3390/genes13122288)
Supplement: Supplementary file 1 [file genes-13-02288-s001.zip › genes-1995496-supplementary.pdf]

Table S1. List of HOPV-A strains included in sequence and phylogenetic analysis.

No	Strain	Origin	Collection Date	Accession No.
1.	A2	Australia- Ref.	1961	NC_038235
2.	ON138-0111A	Canada- prototype	2011	JN257694
3.	Long	USA	1956	M17212
4.	AL19452-2	USA	2000	AF233901
5.	NY20	USA	2000	AF233918
6.	CN2851	Canada	2000	AF233907
7.	SA99V360	South Africa	2001	AF348804
8.	CN2395	Canada	2000	AF233905
9.	MON-4-90	Uruguay	1990	Z33426
10.	Mon-6-97-119	Uruguay	1997	AY571777
11.	TOi2002	Italy	2003	EU025244
12.	SA97D669	South Africa	2001	AF348809
13.	SA98V603	South Africa	2001	AF348807
14.	BJ-F6984-2008.	China	2008	KC297416
15.	RUH-RSVA-2014	Saudi Arabia	2014	KU726070
16.	1844-RSVA-Jeddah-2017	Saudi Arabia	2017	MN434112
17.	1882-RSVA-Jeddah-2017	Saudi Arabia	2017	MN434118
18.	1894-RSVA-Jeddah-2017	Saudi Arabia	2017	MN434119
19.	0155-RSVA-Jeddah-2017	Saudi Arabia	2017	MN434098
20.	Riyadh-1-2014	Saudi Arabia	2014	MH388029
21.	Riyadh-7-2016	Saudi Arabia	2016	MH388030
22.	Riyadh-16-2015	Saudi Arabia	2015	MH388031
23.	Riyadh-16-2016	Saudi Arabia	2016	MH388032
24.	Riyadh-64-2016	Saudi Arabia	2016	MH388033
25.	Riyadh-73-2016	Saudi Arabia	2016	MH388034
26.	Riyadh-79-2016	Saudi Arabia	2016	MH388035
27.	Riyadh-81-2015	Saudi Arabia	2015	MH388036
28.	Riyadh-89-2015	Saudi Arabia	2015	MH388037
29.	Riyadh-112-2015	Saudi Arabia	2015	MH388039
30.	Riyadh-113-2015	Saudi Arabia	2015	MH388040
31.	Riyadh-114-2015	Saudi Arabia	2015	MH388041
32.	Riyadh-116-2015	Saudi Arabia	2015	MH388042
33.	Riyadh 01-2008	Saudi Arabia	2008	JF714705
34.	Riyadh 27-2008	Saudi Arabia	2008	JX131637
35.	Riyadh 38-2008	Saudi Arabia	2008	JX131638
36.	Riyadh 39-2008	Saudi Arabia	2008	JX131639
37.	Riyadh 42-2008	Saudi Arabia	2008	JX131640
38.	Riyadh 83-2009	Saudi Arabia	2009	JX131641
39.	Riyadh 88-2009	Saudi Arabia	2009	JX131642
40.	Riyadh 89-2009	Saudi Arabia	2009	JX131643
41.	Riyadh 91-2009	Saudi Arabia	2009	JF714706
42.	Riyadh 94-2009	Saudi Arabia	2009	JX131644
43.	Riyadh 98-2009	Saudi Arabia	2009	JX131645

Table S2: List of HOPV-B strains included in sequence and phylogenetic analysis

No	Strain	Origin	Collection Date	Accession No.
1.	B1	USA- Ref.	1997	NC_001781
2.	BA4128-99B	Argentina- prototype	1999	AY333364
3.	Riyadh 28-2008	Saudi Arabia	2008	JF714707
4.	Riyadh 52-2008	Saudi Arabia	2008	KC791694
5.	Riyadh 85-2009	Saudi Arabia	2009	KC791695
6.	Riyadh 86-2009	Saudi Arabia	2009	JF714708
7.	Riyadh 133-2009	Saudi Arabia	2009	KC791697
8.	1820-Jeddah-2017	Saudi Arabia	2017	MW019658
9.	1970-Jeddah-2017	Saudi Arabia		MW01966
10.	RUH-RSV_B-42-16	Saudi Arabia	2016	KY711304
11.	RUH-RSVB-2-14	Saudi Arabia	2014	KU726061
12.	CB89-08	Korea	2008	AHQ699308
13.	BA-1607-04	Argentina	2004	DQ227397
14.	NG-004-03	Japan	2003	AB175819
15.	BE-13159-02	Belgium	2002	AY751117
16.	BE-395-85	Belgium	2004	AY751280
17.	BA-3931-99	Argentina	1999	DQ227365
18.	18537	USA	1962	M17213
19.	WV15291	USA	1991	M73542
20.	CH93-9b	USA	1998	AF065251
21.	NY97	USA	2000	AF233932
22.	SA97D934	South Africa	1997	AF348817
23.	NY01	USA	2000	AF233931
24.	CN1839	USA	2000	AF233926
25.	SA0025	South Africa	2001	AF348825
26.	Ken-109-02	Kenya	2002	AY524573
27.	MOZ-202-99	Mozambique	1999	AF309677
28.	SA99V800	South Africa	1999	AF348821
29.	SA0028	South Africa	2001	AF348812
30.	SA98V192	South Africa	1998	AF348811
31.	Cam2009-2145	Cambodia	2009	JN119987
32.	Cam2009-2164	Cambodia	2009	JN119989
